# Patient outcomes, efficiency, and adverse events for elective hip and knee replacement in private and NHS hospitals: a population-based cohort study in England

**DOI:** 10.1016/j.lanepe.2024.100904

**Published:** 2024-04-17

**Authors:** Michael Anderson, Rocco Friebel, Laia Maynou, Ilias Kyriopoulos, Alistair McGuire, Elias Mossialos

**Affiliations:** a Health Organisation, Policy, Economics (HOPE), Centre for Primary Care & Health Services Research, The University of Manchester, United Kingdom; b LSE Health, Department of Health Policy, London School of Economics and Political Science, London, United Kingdom; c Department of Econometrics, Statistics and Applied Economics, Universitat de Barcelona, Barcelona, Spain; d Center for Research in Health and Economics, Universitat Pompeu Fabra, Barcelona, Spain; e Institute of Global Health Innovation, Imperial College London, London, United Kingdom

**Keywords:** Healthcare quality, Private healthcare, Patient safety, Adverse events, Surgery

## Abstract

**Background:**

Since the early 2000s, the National Health Service (NHS) in England has expanded provision of publicly funded care in private hospitals as a strategy to meet growing demand for elective care. This study aims to compare patient outcomes, efficiency and adverse events in private and NHS hospitals when providing elective hip and knee replacement.

**Methods:**

We conducted a population-based cohort study including patients ≥18 years, undergoing a publicly funded elective hip or knee replacement in private and NHS hospitals in England between January 1st 2016 and March 31st 2019. Comparative probability was estimated for three patient outcome measures (in-hospital mortality, emergency readmissions with 28 days, hospital transfers), two efficiency measures (pre-operative length of stay (LOS) >0 day and post-operative LOS >2 days), and four adverse events (hospital-associated infection, adverse drug reactions, pressure ulcers, venous thromboembolism). Probit regression was used to adjust for observable confounding followed by instrumental variable (IV) analyses to also account for unobserved confounding at the patient-level. Propensity score matching was then used as a robustness check.

**Findings:**

Our study sample included 169,232 patients in private hospitals, and 262,659 patients in NHS hospitals. Estimates from probit regression indicated that treatment in private hospital was associated with reduced probability of in-hospital mortality (−0.0009, 95% CI −0.0010, −0.0007), emergency readmissions (−0.0181, 95% CI −0.0191, −0.0172), hospital transfers (−0.0076, 95% CI −0.0084, −0.0068), prolonged post-operative LOS (−0.1174, 95% CI −0.1547, −0.0801), hospital-associated infection (−0.0115, 95% CI −0.0123, −0.0107), adverse drug reactions (−0.0051, 95% CI −0.0056, −0.0046), pressure ulcers (−0.0017, 95% CI −0.0019, −0.0014), and venous thromboembolism (−0.0027, 95% CI −0.0031, −0.0022). IV analyses produced no significant differences between private and NHS hospitals, except for lower probability in private hospitals of hospital-associated infection (−0.0057, 95% CI −0.0081, −0.0032), and greater probability in private hospitals of prolonged post-operative LOS (0.2653, 95% CI 0.1833, 0.3472). Propensity score matching produced similar results to probit regression.

**Interpretation:**

Our findings indicate there is potentially important unobservable confounding at the patient-level between private and NHS hospitals not adjusted for when using probit regression or propensity score matching.

**Funding:**

This research did not receive any dedicated funding.

**Research in context**

**Evidence before this study**

We conducted searches using Medline and EMBASE databases for studies published between January 2000 and December 2023. We used the following search terms: ((independent or private) AND (england OR united kingdom OR britain) AND (hospital OR healthcare or clinic or centre) AND (surg∗) AND (quality OR mortality OR death OR transfer OR readmission OR adverse event OR pressure ulcer OR thromboembolism OR infection OR drug reaction OR length of stay)). We also searched the reference lists of studies identified in our search. Our eligibility criteria were quantitative analyses that focused on differences in patient outcomes, adverse events, and efficiency measures between private and NHS hospitals in England. Most evidence to date indicates that treatment in private hospitals is associated with better outcomes, and higher efficiency than NHS hospitals. One notable exception is a study that used an instrumental variable (IV) approach to account for unobserved confounding at the patient-level and found no difference in the probability of emergency readmissions between private and NHS hospitals.

**Added value of this study**

We extend the approach taken in a previous study and compare results from probit regression, IV analyses, and propensity score matching to examine differences between private and NHS hospitals for a broad range of patient outcomes, adverse events, and efficiency measures. We focus on elective primary hip and knee replacement and replicate the findings of previous studies that only adjust for observable patient-level confounding when using Probit regression. When using an IV approach, we find no evidence of a healthcare quality differential between NHS and private hospitals except for a lower probability of hospital associated infections in private hospitals. Contrary to previous evidence, we also find that treatment in private hospitals is associated with greater post-operative length of stay. To our knowledge, this is the first study to examine differences in the incidence of several, potentially avoidable, adverse events between private and NHS hospitals in England.

**Implications of all the available evidence**

Previous evidence indicating that private hospitals provide higher quality of care than NHS hospitals potentially overlooks unobserved confounding at the patient-level. Improved data collection is needed on aspects of medical complexity such as fitness for surgery and frailty to comprehensively understand differences in case-mix between private and NHS hospitals. Regular and systematic monitoring of healthcare quality in the private healthcare sector is also required.

## Introduction

Since the implementation of market-based healthcare reforms in the England in the mid-2000s,^1^ private hospitals have provided a larger proportion of publicly funded care for several common elective procedures. In 2023, private hospitals conducted approximately 10% of total National Health Service (NHS) funded elective procedures.^2^ For some elective procedures, such as cataract repair, inguinal hernia repair and hip and knee replacement, nearly one out of every three treatments funded by the public sector is conducted in private hospitals.^3^ Irrespective of whether care is privately or publicly funded, there have been concerns regarding the safety and quality of care delivered in private hospitals.^4^, ^5^, ^6^, ^7^ These include concerns regarding lack of oversight and transparency in the reporting of activity and outcome data, as highlighted by the Paterson Inquiry,^4^ and many instances of unsafe practice identified by the Care Quality Commission (CQC), such as inadequate cleanliness and infection control measures, a lack of formal processes to learn from patient safety incidents, and failure to adhere to recommended surgical checklists.^8^

There are two streams of literature that have examined healthcare quality in private hospitals. The first stream uses methods, such as generalised linear modelling or propensity score matching, to adjust for observable confounding at the patient-level and suggests that private hospitals generally have better outcomes and greater efficiency than public hospitals. The largest and most comprehensive study to date from England, by Crothers et al. (2021), analysed nearly half a million operations conducted in private hospitals. Their findings indicated that elective surgery in private hospitals is associated with shorter length of stay and lower emergency readmission rates compared to NHS hospitals.^9^ Similar findings have been produced by other studies from England.^9^, ^10^, ^11^, ^12^, ^13^, ^14^, ^15^ This approach has also been used extensively internationally, with mixed findings in relation to patient outcomes between public and private hospitals.^16^ For example, there is evidence from Germany and Italy that private hospitals have better mortality rates than public hospitals.^17^, ^18^, ^19^ Whereas, in France treatment in private hospitals was associated with poorer mortality rates and higher rates of readmissions that in public hospitals.^20^^,^^21^

The second stream of literature acknowledges the possibility of unobservable confounding at the patient-level. This can arise because confidential contractual arrangements, typically agreed upon at the local level, often stipulate how private hospitals are expected to handle less complicated patients.^22^^,^^23^ Medical complexity is, in part, unobservable as it is often based upon a clinical assessment and not entirely captured by observable patient characteristics such as age, gender, deprivation, and the number of comorbidities.^24^^,^^25^ One study from England, by Moscelli et al. (2018), attempted to overcome this issue by using differential distance between nearest private and NHS hospital as an instrumental variable (IV) and found no significant differences in emergency readmission rates between NHS and private hospitals when analysing a range of high-volume procedures.^26^ IV approaches are widely employed method for causal inference. They help reduce bias by accounting for both unobserved and observable confounding.^27^ Distance to nearest hospital has also been used as an instrument to analyse healthcare quality differences between public and private hospitals in Germany,^28^ Italy,^29^ and Norway.^30^ In all three cases, IV analyses produced different results to those from methods that only adjust for observable confounding demonstrating the influence of unobserved patient confounding when conducting comparative analyses of healthcare quality between public and private hospitals.

This paper aims to build on the approach developed by Moscelli et al. (2018) by analysing a broader range of healthcare quality indicators, including several patient outcomes (in-hospital mortality, emergency readmissions within 28 days, hospital transfers), two efficiency measures (pre-operative length of stay, post-operative length of stay), and four potentially avoidable adverse events (hospital-associated infections, adverse drug reactions, pressure ulcers and venous thromboembolism). Expanding the analysis to account for both unobservable and observable confounding at the patient level will offer additional evidence regarding the quality, efficiency, and outcomes of care in both the NHS and private healthcare sectors in England.

## Methods

### Study design and cohort

We undertook a population-based cohort study, including patients ≥18 years, undergoing a publicly funded elective hip or knee replacement in private and NHS hospitals in England between January 1st 2016 and March 31st 2019. Publicly funded elective care in England is provided by a combination of private and NHS hospitals. Since 2009, patients have choice of both NHS and private providers at the point of referral from GP as a formal right within the NHS constitution.^31^ A full overview of policies that have enabled the provision of publicly funded care in private hospitals over the last two decades is contained in supplementary material (Supplementary Material Section 1). Private hospitals in England include both for-profit and not-for-profit institutions. Independent sector treatment centres (ISTCs) are a specific type of private hospital that specialise in the provision of publicly funded high-volume and low complexity elective care procedures. Whereas conventional private hospitals (i.e. not treatment centres) often undertake both publicly and privately funded care and provide a greater variety of elective care. There are a small number of NHS treatment centres which exclusively treat elective patients. However, most NHS hospitals provide a combination of acute and elective care services. Private hospitals typically do not have access to critical care services,^23^ therefore can decline to treat a publicly funded patient if they deem the patient as higher risk of requiring critical care support in the post-operative period. Both NHS and private hospitals are reimbursed according to the same tariff system according to the Payment by Results (PbR) programme,^32^ which involves fixed activity-based payments for thousands of Healthcare Resource Groups (HRGs) based on average costs for relevant individual procedures or hospital stays.

Patient-level information was obtained from the NHS England Hospital Episode Statistics (HES) database. Primary hip and knee replacements were analysed specifically as they are high-volume procedures in private hospitals. They also have readily available data on Patient Reported Outcome Measures (PROMs) that can be used to adjust for differences in case mix between hospitals. Relevant records according to specific procedural codes for primary hip and knee replacements defined by the National Joint Registry were retrieved.^33^ The full list of these procedural codes is contained in appendices (Supplementary Material Tables S1 and S2). The HES database contains detailed information from pseudonymised patient records for all publicly delivered elective care in England in both NHS and private hospitals. For each patient, information on demographic characteristics, diagnosis information, discharge destination, emergency readmissions, length of stay, and in-hospital death were retrieved. Each patient record was also linked to PROMs data collected through the national NHS England PROMs programme, which is applicable to both NHS and private hospitals.^34^ However, we were unable to link PROMs data beyond March 31st 2018 as this was unavailable to us. The PROMs data were also not available for all patients as completion of PROMs surveys is optional, and therefore PROMs data were only included in supplementary analyses. HES data are structured in finished episodes of care, which are linked to a clinician responsible for a respective aspect of the care pathway. To assess the risk of adverse events during the entirety of the hospital stay, all hospital episodes were combined from day of admission to the day of discharge into hospital spells. Once volumes of hip and knee replacements in each hospital site were calculated, patient episodes were removed from the sample if they were conducted in a hospital site that undertook less than 30 elective hip or knee replacements during our period of analysis. Patient episodes were also excluded if they were coded as a HRG conducted less than 10 times in private hospitals during our period of analysis.

### Study outcomes

We analysed three patient outcome measures (in-hospital mortality, emergency readmissions within 28 days, and inter-hospital transfer), two efficiency measures (pre-operative and post-operative length of stay), and four common and potentially preventable adverse events (hospital-associated infections, adverse drug reactions, pressure ulcers and venous thromboembolism). Identification of adverse events was based on relevant diagnosis codes according to the International Statistical Classification of Diseases and Related Health Problems, 10th edition (ICD-10) (Supplementary Material Table S3).^f^ Patients who died during their hospital stay or were transferred to another hospital were identified based on the record of the discharge method. Emergency readmissions were identified by using unique patient identifiers and defined as non-elective admissions to any hospital within 28 days of discharge. Pre-operative length of stay was calculated as the difference between day of admission and day of surgical procedure, and post-operative length of stay was calculated as the difference between day of surgical procedure and day of discharge. Separating length of stay into pre-operative and post-operative is an approach previously used to analyse the impact of competition on efficiency in private and NHS hospitals.^35^ We coded extended pre-operative length as stay as longer than 0 days, as this indicates the patient was admitted the night before surgery. We coded extended post-operative length of stay as longer than 2 days, to align with NHS England Getting It Right First Time (GIRFT) guidance that recommends discharge should be considered 48 h following elective primary hip and knee replacement.^36^

### Covariates

The HES database also includes information on patient characteristics, including age, gender, deprivation, and comorbidities which we draw on as covariates. Age was coded as a categorical variable into four groups (18–40 years old, 40–60 years old, 60–80 years old, older than 80 years old). Deprivation is recorded according to the English Indices of Multiple Deprivation (IMD) 2015,^37^ which is calculated by ranking the 32,844 small areas in England from most deprived to least deprived and dividing them into equal groups based upon seven domains including income, employment, education, health and disability, crime, barriers to housing and services, and living environment. We split the IMD index into quintiles, with quintile 1 representing the most deprived and quintile 5 representing the least deprived. The latest version of the Charlson Comorbidity Index (CCI) that draws upon ICD-10 codes was used as a measure for patient complexity based on the number of comorbidities recorded in each admission.^38^ We coded the CCI as a categorical variable based on the number of comorbidities, ranging from 1 to more than 6. The specific PROMs included in this analysis are the pre-operative Oxford Hip Score (OHS),^39^ and the Oxford Knee Score (OKS),^40^ categorised according to score 0–19 (severe arthritis), score 20–29 (moderate to severe arthritis), score 30–39 (mild to moderate arthritis), and score 40–48 (satisfactory joint function). Pre-operative PROM scores were also coded as a categorical variable and included as covariates to adjust for differences in disease-specific disability between patients at baseline. The extent of missing data for each covariate is listed within supplementary material (Supplementary Material Table S4), with generally low levels of missingness noted for each covariate.

### Statistical analysis

Two main approaches were used to examine differences in patient outcomes, adverse events, and efficiency between private and NHS hospitals. First, probit regression was used to adjust for observable confounding between private and NHS hospitals. Second, differential distance between nearest NHS and private hospitals was used as an instrumental variable (IV) to also account for unobserved confounding at the patient-level. All analysed were conducted using STATA v17, and the relevant code used for all analyses is contained in a publicly available repository (https://github.com/mikeuk2024/NHS-and-private-hospital-analysis/blob/main/2024Lancetdo_09_03.do).

#### Probit regression

Probit regression was used to estimate the comparative probability of experiencing different adverse events and outcome measures in private and NHS hospitals, and the association between treatment in private hospitals and different efficiency measures. Analyses were undertaken using the “probit” STATA command.^41^ To account for week versus weekend and seasonal variation, two time-variables were added (*i.e*., weekdays versus weekend, and winter versus non-winter period). Binary variables for each year of the analysis were included to difference out any year-to-year variation. Robust standard errors were used, clustered at the HRG level.

The following specification was estimated [1]:

[1] $$\mathrm{Pr}\left(Y_{ij}=1\;|\;H,X,Z\right)=\Phi \;\left(\alpha_{i}+\delta H_{ij}+\beta X_{ij}+\gamma Z_{ij}\right)$$

Where Φ(·) is the cumulative standard normal distribution function, *Y*_*ij*_ indicates the dependent variable, whether the patient *i* experienced an outcome, efficiency measure, or adverse event in hospital *j*; $\alpha_{i}$ is the fixed effects of relevant HRGs,^g^ $H_{ij}$ is a binary variable that is equal to 1 if hospital *j* is a private hospital and 0 if a NHS hospital; *X*_*ij*_ is a vector of patient characteristics (*i.e*., age, gender, deprivation, CCI), *Z*_*ij*_ denotes the time-variables (*i.e*., year, weekdays versus weekend, and winter period). The coefficient of interest is the difference in the probability of an adverse event, outcome, or efficiency measure following an elective hip or knee replacement in a private hospital compared to an NHS hospital.

#### Instrumental variable (IV) analysis

Our IV analyses used differential distance between NHS and private hospitals from the centroid of a patient's Lower Layer Super Output Areas as an instrument for hospital choice. This instrument was also adopted by Moscelli et al. (2018) in their narrower study, and has been used by several other studies that analyse the association between hospital ownership and quality of care.^42^, ^43^, ^44^, ^45^, ^46^ Several previous studies have also included further specifications of IVs to assess the robustness of their results,^42^, ^43^, ^44^, ^45^, ^46^ using the respective distances to the nearest NHS and private hospital as two further instruments. We adopt a similar robustness approach. Our analyses used the “ivprobit” STATA command.^47^

The equation for the first stage IV regression is:

[2] $$H_{i}=\alpha_{i}\;+\;\emptyset D_{i}\;+\;\beta X_{i}\;+\;\gamma Z_{i}\;+\;e_{i}$$

where *H*_*i*_ indicates the dependent variable, whether the patient *i* is treated in a private or NHS hospital; $\alpha_{i}$ is the fixed effects of relevant HRGs, $D_{i}$ is the instrument, specifically differential distance between the nearest NHS and private hospital for patient *i*; *X*_*i*_ is a vector of patient characteristics (*i.e.,* age, gender, deprivation, CCI), *Z*_*i*_ denotes the time-variables (*i.e.,* year, weekdays versus weekend, and winter period).

This approach relies on having an instrument that meets the following assumptions: the instrument is associated with the treatment exposure (e.g. choice of hospital); the instrument should be randomized with respect to the outcome and treatment variables; the instrument has no relationship to the outcome of interest except through the treatment exposure itself; and the instrument cannot increase the exposure or treatment level for some individuals and decrease it for others.^27^^,^^48^ There are logical reasons why differential distance to NHS and private hospitals is a good instrument that meets these assumptions. First, we would expect distance to hospital to be correlated with choice of hospital as surveys have indicated that patients prioritise geographical location as the single most important factor when choosing healthcare providers in England.^49^ Moreover, analyses have indicated that traditional measures of healthcare quality such as mortality and emergency readmission rates have little impact on demand for hospital care in England.^50^ Second, the instrument should be randomised in relation to the treatment and outcome as it does not seem plausible that quality of care for elective care significantly impacts patient decisions about where to live as this would require patients to prospectively plan what treatments they require and anticipate the future quality of care for these treatments in different hospitals.^51^^,^^52^ Third, we would not expect differential distance to nearest NHS and private hospital to increase likelihood of choosing a private hospital for some patients and decrease this likelihood for other patients. However, we acknowledge that the strength of the relationship between differential distance to nearest NHS and private hospital and choice of hospital may vary between patient groups, and we take account of this to what extent is possible by using HRG fixed effects and adjusting for observable patient confounders such as age, gender, deprivation, and number of comorbidities.

#### Supplementary analyses

Several further supplementary analyses were conducted to assess the robustness of the results to different specifications and subgroups of populations. First, patients’ pre-operative PROM scores were included as a patient characteristic within regression models to ascertain if this significantly changed the results. Second, the Probit regression and IV models were repeated for private and NHS hospitals in the Greater London area only, as higher volumes of elective care occur in private hospitals in London,^53^ and there may be less variation in quality of care than in other regions. Third, NHS treatment centres were compared against Independent Sector Treatment Centres (ISTCs), and NHS acute hospitals compared against private hospitals (excluding ISTCs). The rationale for these comparisons was that NHS treatment centres only deliver elective care and therefore are more directly comparable to ISTCs than NHS acute hospitals. Fourth, NHS hospitals were compared against for-profit private hospitals, and against not-for-profit hospitals. We undertook this analysis as it is possible that for-profit private hospitals may prioritise cost-savings over quality of care, or conversely may provide better quality of care if they perceive this as an important determinant of demand for their healthcare services.^26^

Finally, propensity score matching was used as a robustness check to examine if this approach produced different results to our IV and probit regression analyses. In our case, one-to-one nearest neighbour matching with replacement was used, which involves matching each treatment observation to a single nearest neighbour in the control group according to their propensity score. Propensity score matching was performed for patients undergoing hip and knee replacement separately, and results expressed as the average treatment effect on treated (ATT) of treatment in a private versus NHS hospital for our outcomes of interest. We first estimated propensity scores based on the patient's age, CCI score, and IMD quintile. When then repeated the propensity score matching also using pre-operative PROM scores. A caliper distance of 0.01 was used, which is the predefined width by which propensity scores can differ for any one match.^54^ To calculate confidence intervals, bootstrapping was performed with 1000 iterations. The quality of covariate balancing was assessed before and after matching and reported in supplementary tables. Our analyses used the “psmatch2” STATA command.^55^

### Role of the funding source

This research did not receive any dedicated funding.

## Results

### Descriptive statistics

The study sample included a total of 75,891 hip replacements and 93,341 knee replacements undertaken in private hospitals, and 166,925 hip replacements and 195,734 knee replacements undertaken in NHS hospitals. Our study sample were treated in 310 NHS hospitals, and 185 private hospitals. We report the patient volumes in NHS and private hospitals according to their classification as treatment centres and financial status in supplementary material (Supplementary Table S5). The sample was concentrated within seven HRGs, which account for 99.71% of the total sample (Supplementary Material Table S6). Patients treated in NHS hospitals were, on average, older, more deprived, and had a higher number of comorbidities. Patients undergoing treatment in private hospitals had significantly better outcomes than those treated in NHS hospitals, both in terms of in-hospital mortality, emergency readmissions, and hospital transfers in these unstandardised descriptives. The prevalence of all adverse events was lower in private hospitals compared to NHS hospitals, with the largest difference in prevalence of hospital-associated infections (Table 1).

**Table 1:** Descriptive statistics of patient characteristics and outcomes for elective hip and knee replacements in NHS and private hospitals.

	NHS			Private		
	362,659 (68.18%)			169,232 (31.82%)		
	No	Mean (SD)	Median (IQR)	No	Mean (SD)	Median (IQR)
**Outcomes**						
In-hospital mortality (=1)	214	0.0006 (0.0243)	0 (0–0)	<8	<0.0001 (0.0064)	0 (0–0)
Emergency readmissions (=1)	26,125	0.0720 (0.2586)	0 (0–0)	8332	0.0492 (0.2164)	0 (0–0)
Hospital transfers (=1)	3121	0.0086 (0.0924)	0 (0–0)	310	0.0018 (0.0428)	0 (0–0)
**LOS**						
Pre-op LOS (days)	362,659	0.04578 (0.7038)	0 (0–0)	169,232	0.1504 (0.6979)	0 (0–0)
Pre-op LOS >0 days	362,659	0.0235 (0.1515)	0 (0–0)	169,232	0.0586 (0.2348)	0 (0–0)
Post-op LOS (days)	362,659	4.2190 (3.6383)	3 (3–5)	169,232	2.8096 (1.4483)	3 (2–3)
Post-op LOS >2 days	362,659	0.7571 (0.4288)	1 (1–1)	169,232	0.6179 (0.4859)	1 (0–1)
**Adverse events**						
HAI (=1)	2712	0.0075 (0.0861)	0 (0–0)	74	0.0004 (0.0209)	0 (0–0)
Adverse drug reaction (=1)	2477	0.0068 (0.0824)	0 (0–0)	368	0.0022 (0.0466)	0 (0–0)
Pressure ulcer (=1)	816	0.0022 (0.0474)	0 (0–0)	100	0.0006 (0.0243)	0 (0–0)
Venous thromboembolism (=1)	1348	0.0037 (0.6085)	0 (0–0)	209	0.0012 (0.0351)	0 (0–0)
**Patient characteristics**						
Female (=1)	214,301	0.5851 (0.4927)	0 (0–1)	95,269	0.5753 (0.4943)	0 (0–1)
Age (years)	362,659	69.4951 (11.1504)	70 (63–77)	169,232	68.8382 (9.6287)	70 (63–76)
18–40 years	1029			118		
41–60 years	2651			536		
61–80 years	301,852			149,388		
>80 years	57,127			19,190		
IMD (quintile)		3.1299 (1.4053)	3 (2–4)		3.3528 (1.4052)	4 (2–5)
1	62,987			24,525		
2	65,221			25,910		
3	68,599			28,941		
4	81,996			44,413		
5	77,750			45,056		
CCI (No of diagnoses)		0.6573 (0.9891)	0 (0–1)		0.4339 (0.7747)	0 (0–1)
0	212,685			117,753		
1	93,618			35,668		
2	35,271			11,129		
3	13,963			3564		
4	4577			857		
5	1250			176		
6+	1295			85		
Weekdays discharge (=1)	273,029	0.7529 (0.4314)	0 (0–1)	117,159	0.6923 (0.4615)	0 (0–1)
Winter discharge (=1)	120,954	0.3335 (0.4715)	0 (0–1)	61,145	0.3613 (0.4804)	0 (0–1)
**PROMs**						
Participation (=1)	159,078	0.6351 (0.4814)	0 (0–1)	72,360	0.6241 (0.4844)	0 (0–1)
Pre-operative hip/knee score	159,078	17.2339 (7.9962)	17 (11–23)	72,360	19.7022 (7.9348)	19 (14–25)

LOS, length of stay; HAI, healthcare-associated infection; IMD, index of multiple deprivation (quintile 1 = most deprived, quintile 5 = least deprived), CCI, Charlson Comorbidity Index; PROMs, patient-reported outcome measures.

### Primary analysis

The results of the Probit regression models are outlined in Table 2 (Model 1 and 2). The inclusion of observable patient-level confounders within the regression model slightly reduced the size of the co-efficient that represented differences in patient outcomes, efficiency, and adverse events between private and NHS hospitals (Model 2). Treatment in private hospital was associated with a significantly reduced probability of in-hospital mortality (−0.0009, 95% CI −0.0010, −0.0007), emergency readmission (−0.0181, 95% CI −0.0191, −0.0172) and hospital transfer (−0.0076, 95% CI −0.0084, −0.0068). Treatment in a private hospital was associated with significantly increased probability of extended pre-operative length of stay (0.319, 95% CI 0.0048, 0.0589) but significantly reduced probability of extended post-operative length of stay (−0.1174, 95% CI −0.1547, −0.0801). The probability of all adverse events was also significantly lower in private hospitals, including for hospital-associated infection (−0.0115, 95% CI −0.0123, −0.0107), adverse drug reaction (−0.0051, 95% CI −0.0056, −0.0046), pressure ulcer (−0.0017, 95% CI −0.0019, −0.0014), and venous thromboembolism (−0.0027, 95% CI −0.0031, −0.0022).

**Table 2:** Results of probit regression and instrumental variable analyses.

	Probit regression with no case-mix adjustment (1)	Probit regression with case-mix adjustment (2)	Instrumental variable analyses (3)
In-hospital mortality	−0.0010 (−0.0012, −0.0009)	−0.0009 (−0.0010, −0.0007)	−0.0004 (−0.0010, 0.0002)
p value	<0.0001	<0.0001	0.2407
R^2^:	0.3035	0.0395	0.1729
Endog test p value:			0.1691
Emergency	−0.0230 (−0.0237, −0.0224)	−0.0181 (−0.0191, −0.0172)	−0.0060 (−0.0190, 0.0070)
Readmission			
p value	<0.0001	<0.0001	0.3660
R^2^:	0.6655	0.1485	0.0625
Endog test p value:			0.0175
Hospital transfer	−0.0090 (−0.0097, −0.0083)	−0.0076 (−0.0084, −0.0068)	0.0046 (−0.0076, 0.0169)
p value	<0.0001	<0.0001	0.4583
R^2^:	0.7241	0.1715	0.0546
Endog test p value:			<0.0001
Pre-op LOS >0 day	0.0304 (0.0029, 0.0579)	0.0319 (0.0048, 0.0589)	0.0464 (−0.0140, 0.1069)
p value	0.0301	0.0210	0.1322
R^2^:	0.2548	0.2398	0.4051
Endog test p value:			<0.0001
Post-op LOS >2 days	−0.1325 (−0.1694, −0.0956)	−0.1174 (−0.1547, −0.0801)	0.2653 (0.1833, 0.3472)
p value	<0.0001	<0.0001	<0.0001
R^2^:	0.6764	0.3020	0.5068
Endog test p value:			<0.0001
HAI	−0.0126 (−0.0134, −0.0118)	−0.0115 (−0.0123, −0.0107)	−0.0057 (−0.0081, −0.0032)
P value	<0.0001	<0.0001	<0.0001
R^2^	0.1845	0.1255	0.4762
Endog test p value:			0.0024
Adverse drug reaction	−0.0058 (−0.0063, −0.0053)	−0.0051 (−0.0056, −0.0046)	0.0014 (−0.0031, 0.0059)
p value	<0.0001	<0.0001	0.5428
R^2^	0.8211	0.3656	0.0163
Endog test p value:			0.0002
Pressure ulcer	−0.0021 (−0.0023, −0.0018)	−0.0017 (−0.0019, −0.0014)	−0.0005 (−0.0015, 0.0006)
p value	<0.0001	<0.0001	0.3750
R^2^	0.3354	0.0797	0.0558
Endog test p value:			0.3179
Venous thrombo-embolism	−0.0030 (−0.0034, −0.0025)	−0.0027 (−0.0031, −0.0022)	−0.0002 (−0.0016, 0.0011)
p value	<0.0001	<0.0001	0.7482
R^2^	0.5343	0.3460	0.0420
Endog test p value:			0.0413
1st-stage F stat:			9404.40
Observations:	531,891	525,363	525,361

Endog test, Hausman endogeneity test; HAI, healthcare-associated infection; LOS, length of stay. 95% Confidence Intervals are in parentheses

In contrast to the Probit regression results, the IV model produced no significant differences in probability of in-hospital mortality (−0.0004, 95% CI −0.0010, 0.0002), emergency readmission (−0.0060, 95% CI −0.0190, 0.0070) and hospital transfer (0.0046, 95% CI −0.0076, 0.0169), extended pre-operative length of stay (0.0464, 95% CI −0.0140, 0.1069), adverse drug reaction (0.0014, 95% CI −0.0031, 0.0059), pressure ulcer (−0.0005, 95% CI −0.0015, 0.0006), and venous thromboembolism (−0.0002, 95% CI −0.0016, 0.0011). The only statistically significant findings were lower probability in private hospitals of hospital-associated infection (−0.0057, 95% CI −0.0081, −0.0032), and greater probability in private hospitals of prolonged post-operative length of stay (0.2653, 95% CI 0.1833, 0.3472). The F-statistic was 9404.40, indicating that differential distance between nearest NHS and private hospital was a good instrument for treatment in private hospital. Focusing on the results of the first stage IV regression (Supplementary Material Table S7), differential distance between nearest NHS and private hospital was also strongly correlated with choice of private hospital. For every 1 km closer that the nearest private hospital is located relative to the nearest NHS hospital, the probability that a patient will be treated in a private hospital increased by 0.007 (95% CI 0.007, 0.007). The Hausman endogeneity test was passed for all indicators except for probability of in-hospital mortality (p value = 0.1691), and pressure ulcer (p value = 0.3179).

As a robustness check, distance to nearest private and NHS hospital were used as two separate instruments for hospital choice (Supplementary Material Table S8). However, the Sargan-Hansen overidentification test rejected the validity of the instruments for all patient outcomes, efficiency measures and adverse events, indicating that this model was incorrectly specified.^56^^,^^57^ For this reason, this specification of the IV analysis was not repeated in any of the further supplementary analyses.

### Supplementary analyses

As PROMs data were only available until March 31st 2018, including PROMs as a patient-level covariate resulted in a different sample size than our primary analysis that used data up to March 31st 2019. The results produced were broadly similar to the primary analyses with some exceptions when undertaking the IV analyses (Supplementary Material Table S9). These included treatment in private hospital associated with increased probability of emergency readmission (0.0117, 95% CI 0.0043, 0.0190), and pre-operative LOS greater than 0 days (0.2943, 95% CI 0.1894, 0.3993).

Restricting analysis to hospitals exclusively located in the Greater London produced similar results to our primary analysis (Supplementary Material Table S10), except that probit regressions did not produce significant findings for probability of in-hospital mortality, pressure ulcers and venous thromboembolism. However, this is likely to reflect the smaller sample size and how these are rare events. Comparing ISTCs to NHS treatment centres, and private hospitals (excluding ISTCs) to NHS hospitals (excluding NHS treatment centres) also produced similar results to our primary analysis (Supplementary Material Tables S11 and S12). There were also no major differences in findings between our primary analysis when analysing private hospitals based on whether they were for-profit or not-for-profit (Supplementary Material Tables S13 and S14). The only exception was no significant difference in probability in hospital-associated infection between NHS and not-for-profit hospitals. However, this is likely to reflect how the sample size was reduced substantially as most private hospitals are for-profit. Propensity score matching produced similar results to probit regression in our primary analysis (Supplementary Table S15), with no notable differences in results when focusing specifically on primary hip or knee replacement. Inclusion of pre-operative PROM scores within the propensity score matching also produced similar results to probit regression (Supplementary Table S17), with the exception that the ATT for in-hospital mortality was not statistically significant. The quality of covariate matching following propensity score matching was generally high (Supplementary Tables S16 and S18).

## Discussion

This analysis provides a comprehensive comparative assessment of patient outcomes, efficiency measures and adverse events in private and NHS hospitals for patients undergoing elective hip and knee replacement in England. Patients treated in private hospitals were less complex, possibly reflecting the preferential contractual arrangements between the NHS and private providers or preferences of patients and clinicians. Using Probit regression to adjust for observable confounding, treatment in private hospitals was associated with a significantly reduced probability of in-hospital mortality, emergency readmission, hospital transfer and several adverse events. Treatment in private hospitals was also associated with longer pre-operative length of stay and shorter post-operative length of stay. In contrast, using differential distance between nearest NHS and private hospital as an IV for hospital choice to adjust for both observable and unobservable confounding at the patient-level, we find there are no significant differences in the probability of most patient outcomes or adverse event between private and NHS hospitals. The only exception was a lower probability of hospital associated infection in private hospitals. We also find no significant differences in pre-operative length of stay, but treatment in private hospital was in fact associated with increased post-operative length of stay.

There are several strengths to this analysis. First, a broad spectrum of healthcare quality indicators was analysed allowing greater inference to be gained in analysing differences across the NHS and private sectors. In contrast, other studies analysed either one or only a few healthcare quality indicators.^10^^,^^26^ To our knowledge, this is also the first study to compare the prevalence and probability of several, potentially avoidable, adverse events in private and NHS hospitals in England. Second, several supplementary analyses were conducted, including hospitals subcategorised according to their status as a treatment centre and their financial objectives giving greater robustness to the results. Third, arguably the most significant strength of this analysis is the application of methods that take account of both observable and unobservable confounders at the patient-level between NHS and private hospitals. This is important as it is known that the case-mix varies significantly between private and NHS hospitals,^22^ and that the scope for potential unobserved confounding at the patient-level is high due to confidential contractual arrangements agreed at the local level that typically specify how private hospitals are expected to treat less complicated patients.^22^^,^^23^

However, there are also limitations to this analysis. First, there are hospital and workforce factors known to influence outcomes, efficiency and adverse events that are not analysed because such data are not available for private hospitals in England. From the hospital perspective, important factors include the presence of critical care facilities,^58^^,^^59^ waiting times, and bed occupancy.^60^^,^^61^ From the workforce perspective, important factors include surgical experience,^62^^,^^63^ vacancy rates,^64^ and nurse-to-patient ratios.^65^ Second, Hausman endogeneity tests were failed for two outcome indicators (in-hospital mortality and pressure ulcers) following our IV analyses indicating there is an element of bias with these co-efficients. However, Moscelli et al. (2018) argue that the direction of this bias is known as private hospitals can select healthier patients and biased results will overestimate any quality-of-care gains from treatment in a private hospital.^26^ As a result, we can still be confident that quality of care for elective hip and knee replacements in NHS hospitals is at least as good as in private hospitals for these two outcome indicators as these coefficients indicate there are no significant differences in quality of care. Third, our identification of adverse events is reliant upon diagnostic coding in hospital administrative records. Therefore, our analyses do not capture adverse events that were either not coded or experienced following discharge from hospital. It is also possible our findings have been influenced by differences in coding practices between NHS and private hospitals. However, we can be reassured by the nearly two decades’ experience that private hospitals have in supplying admitted patient care data to NHS England. Moreover, private hospitals typically outsource their clinical coding to the same private consultancy companies that support NHS hospitals in England with their clinical coding audits.^66^, ^67^, ^68^ Fourth, our analysis focuses exclusively on elective hip and knee replacement in private and NHS hospitals. Further analysis is required to establish to what extent different methods for comparative analysis produce different results for other procedures commonly undertaken in private hospitals in England. Fifth, our analysis is focused specifically on the context for elective care provision in NHS and private hospitals within England. However, there are many healthcare systems that use a mixed economy of public and private providers to deliver publicly funded elective care that could replicate this analysis using similar healthcare administrative datasets from their own countries.^16^ Finally, our analysis includes data until March 31st 2019 and more recent data would provide insights into differences in healthcare quality between the NHS and private hospitals during the COVID-19 pandemic and beyond. This decision was made as patterns in elective care provision in both NHS and private hospitals changed dramatically during the COVID-19 pandemic to prioritise more urgent elective cases and there was substantial regional variation in the continued provision of publicly funded care in private hospitals.^69^^,^^70^ Future research can examine how these changes in elective care delivery in private hospitals have influenced healthcare quality.

Our analyses have several implications for policy and future research. Despite literature suggesting that NHS hospitals provide poorer quality of care than private hospitals, our IV analyses find no evidence of a healthcare quality differential between NHS and private hospitals except for lower probability of hospital associated infections in private hospitals. This means the narrative that private hospitals provide a safer environment to provide elective care than in NHS hospitals may not be correct, and we argue there is a need for more systematic and regular monitoring of patient outcomes, efficiency, and adverse events in the private health care sector. For such data collection exercises to comprehensively reflect differences between NHS and private hospitals, they will need coupled with improved data collection for organisational and workforce characteristics, and operational research regarding different patient pathways to help understand some of the observed disparities in quality of care between the two sectors. This will allow more comprehensive analysis of healthcare quality across private hospitals according to differences in hospital size, surgeon experience, staffing levels, and facilities available. We also expose significant differences in case-mix between private and NHS hospitals. These differences in case-mix between private and NHS hospitals indicate that private hospitals may be engaging in cream-skimming to avoid costly patients. As we find evidence of unobserved confounding at the patient-level, it may be that current reimbursement tariffs are not fit for purpose to account for differences in patient case-mix between private and NHS hospitals. Therefore, there is a need for research to examine the financial implications for NHS hospitals of increased delivery of publicly funded care in private hospitals and potential options for adjustments to reimbursement to account for any advantages private hospitals may have in the market for publicly funded elective care.

## Contributors

MA undertook the analysis and drafted the manuscript. All other authors were collaboratively involved in the design of the analysis and provided comments and edits to iterative versions of the manuscript.

## Data sharing statement

NHS England Hospital Episode Statistics can be accessed following an application to the NHS England Data Access Request Service (DARS).

## Declaration of interests

MA declares he has received consultancy fees from the Private Healthcare Information Network that was unrelated to this work. The other authors have no relevant conflicts of interest to declare related to this manuscript.

## Footnotes

f The selection of codes followed those used in previous studies as they have shown high validity and specificity in the detection of adverse events from electronic health records.^35^, ^36^, ^37^, ^38^ The relevant inclusion and exclusion criteria set out in the Patient Safety Indicators (PSI) of the US Agency for Healthcare Research and Quality (AHRQ) were used to identify hospital-associated adverse events from administrative patient records. The AHRQ PSI indicators have been translated and validated for use in England.^39^ The coding for adverse drug reactions (which is not a PSI developed by AHRQ) was retrieved from a much cited manuscript focused on the prevalence of adverse drug reactions in an Australian hospital dataset,^40^ which has since been applied to HES in England.^35^

g Fixed effects were not used at the hospital level, as this was attempted and the relevant variable that distinguished between private and NHS hospitals was dropped from the regression model as it was constant within groups of analysis. Fixed effects were used at Healthcare Resource Group (HRG) level to reflect differences in surgical complexity between different HRGs following the approach used in Moscelli et al., 2018. HRGs are fixed activity-based payments based on average costs for relevant individual procedures or hospital stays. HRGs are calculated according to combinations of procedural codes, diagnostic codes, and age. Length of stay influences the tariff that hospitals receive at a decreasing margin to remove incentives to prolong hospital stays.
