# Supplementary material for: Patient outcomes, efficiency, and adverse events for elective hip and knee replacement in private and NHS hospitals: a population-based cohort study in England
Source: Lancet Reg Health Eur. 2024 Apr 17;40:100904. doi: 10.1016/j.lanepe.2024.100904 (PMC11047790; doi:10.1016/j.lanepe.2024.100904)
Supplement: Supplementary Material [file mmc1.docx]

**Supplementary Material Table of Contents**

[1. Background of publicly funded elective care in private hospitals in England 2](#_Toc162346986)

[1.1. Healthcare market policies from the 2000s onwards 3](#_Toc162346987)

[1.1.1. Patient choice of provider 4](#_Toc162346988)

[1.1.2. Independent sector treatment centres 4](#_Toc162346989)

[1.1.3. Activity-based payments 5](#_Toc162346990)

[1.1.4. Greater emphasis on targets 6](#_Toc162346991)

[1.1.5. Strengthened regulation 6](#_Toc162346992)

[1.1.6. Structural changes to promote an internal market for healthcare 7](#_Toc162346993)

[1.1.7. References 7](#_Toc162346994)

[2. Supplementary Tables 11](#_Toc162346995)

[Table 1: Hip primary OPCS procedure codes and descriptions 11](#_Toc162346996)

[Table 2: Knee primary OPCS procedure codes and descriptions 13](#_Toc162346997)

[Table 3: ICD-10 codes used for identification of adverse events 14](#_Toc162346998)

[Table 4: Missing data 15](#_Toc162346999)

[Table 5: Hospital Type and Volume between 2016-2019 16](#_Toc162347000)

[Table 6: HRGs for NHS and Private hospitals 17](#_Toc162347001)

[Table 7: Results of first stage regression for 2SLS IV analysis 18](#_Toc162347002)

[Table 9: Results of OLS and IV models for all NHS and private hospitals when including PROMs as covariate 20](#_Toc162347003)

[Table 10: Results of OLS and IV models for all NHS and private hospitals for London subanalysis 21](#_Toc162347004)

[Table 11: Results of OLS and IV models for NHS treatment centres versus Independent Sector Treatment Centres (ISTCs) 22](#_Toc162347005)

[Table 12: Results of OLS and IV models for NHS Acute Hospital versus Private Hospitals (excluding ISTCs) 23](#_Toc162347006)

[Table 13: Results of OLS and IV Models for NHS hospitals versus For-Profit Private Hospitals 24](#_Toc162347007)

[Table 14: Results of OLS and IV Models for NHS hospitals versus Not-For-Profit Private Hospitals 25](#_Toc162347008)

[Table 15: Results of nearest neighbour propensity score matching for primary analysis 26](#_Toc162347009)

[Table 16: Quality of covariate matching for primary analysis 26](#_Toc162347010)

[Table 17: Results of nearest neighbour propensity score matching when including PROMs 27](#_Toc162347011)

[Table 18: Quality of covariate matching when including PROMs 27](#_Toc162347012)

# Background of publicly funded elective care in private hospitals in England

The purpose of this section is to provide context and background regarding the development of provision of publicly funded elective care in private hospitals in England.

In England, the Department of Health & Social Care (DHSC) allocates funds to NHS England, which then distributes funds to local commissioning bodies known as Integrated Care Boards (ICBs) (prior to July 2022 these were known as clinical commissioning groups, CCG), as well as to specialist and primary care services (Figure 1). The DHSC makes further allocations of funds to arm’s-length bodies, such as the National Institute for Health and Care Excellence (NICE),^1^ the Care Quality Commission (CQC),^2^ and NHS Resolution.^3^

*Figure 1: Funding flows in the health and social care system, 2019/20*


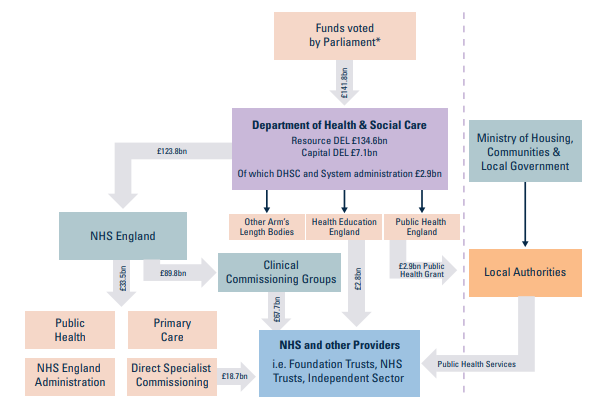


Local commissioning bodies receive block grants from NHS England that are calculated according to local needs and market forces, and subsequently contract for emergency and elective hospital services, as well as community and mental health services for their respective local populations. ICBs may contract services from either NHS or private hospitals, and since 2009 patients have a right to choose where they receive elective care services. As a result, there has been a growing expansion of publicly funded care in private hospitals in England, and in 2019/20 private hospitals undertook approximately 6% of total publicly funded elective episodes (Figure 2).

*Figure 2: Share of publicly funded elective treated in the private healthcare sector, 2003/04 to 2020/21*


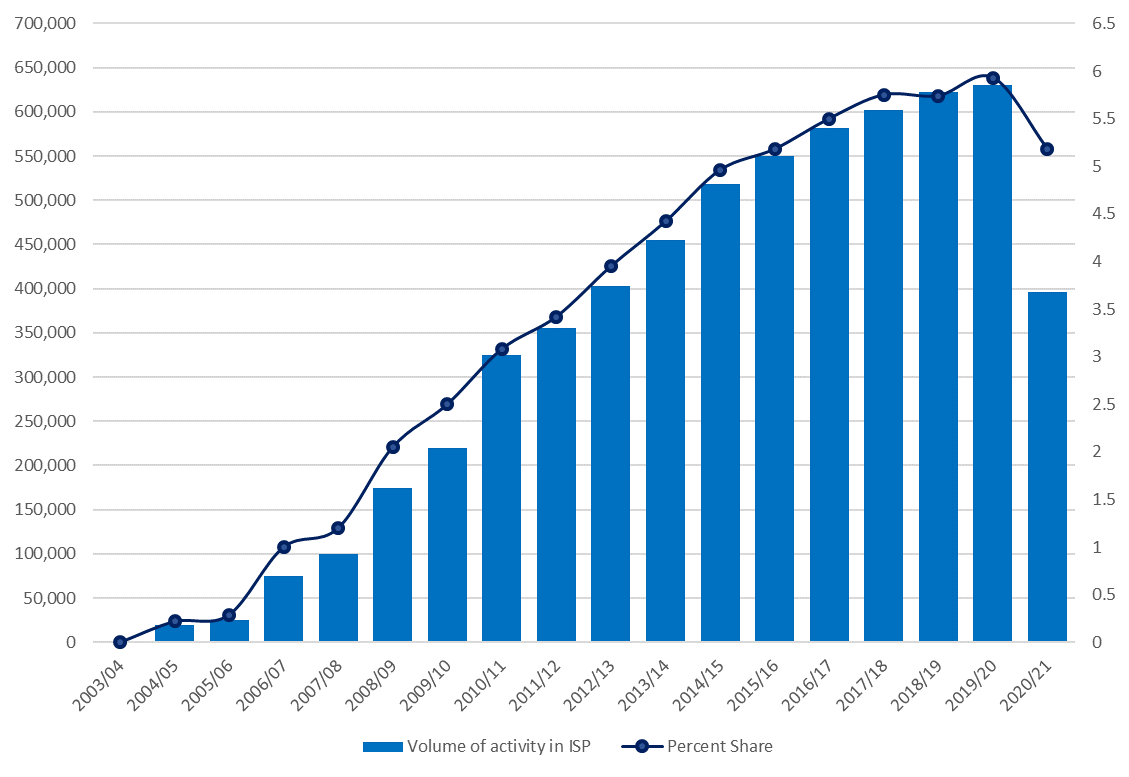


Source: Reproduced from ^4^

## Healthcare market policies from the 2000s onwards

In the late 1990s, waiting lists for many types of elective care were approaching over a year, ^5^ and health spending per capita was significantly below the EU average.^6^ Over the following decade, the incumbent Labour government made a commitment to significantly increase health spending to a sum closer to the EU average and introduced policies to facilitate patient choice and promote greater competition between healthcare providers. The rationale was that this would improve healthcare quality, efficiency, and responsiveness in the public sector.^7^ Collectively, these policies facilitated the rapid expansion of provision of publicly funded elective care services in private hospitals. In this section, I review key policies and reforms that drove these changes.

- - 1. Patient choice of provider

Enabling patient choice for their healthcare provider was a key component of health policy promoted by the Labour government throughout the 2000s. In 2000, the NHS Plan outlined policies that patients should be able to choose the times and dates of their hospital appointments.^8^ In 2002, the associated implementation plan titled “Delivering the NHS Plan: Next steps on investment, next steps on reform”, was more specific and included a commitment to offer patients the choice of an alternative provider if they could not be treated within six months by the NHS.^9^ This was originally framed as a way of reducing waiting times but in subsequent years patient choice was increasingly promoted as a mechanism to facilitate competition between healthcare providers and to create incentives to improve healthcare quality. In 2006, the NHS in England introduced a policy of choice of provider at point of referral in primary care. The intention was that all patients were to be offered a choice of several providers at the point of referral, including choice of private healthcare providers. In 2009, patient choice of healthcare provider was embedded as a formal right for all patients within the NHS Constitution.^10^ The “free choice” policy was maintained by the subsequent Coalition government, which introduced the “Any Qualified Provider (AQP)” contractual system in 2012 that expanded the range of services that private healthcare providers could compete for with NHS providers.^11^ Despite these policy changes, patients continue to have limited choice of healthcare providers unless they are willing to travel large distances. For most patients this is not the case, demonstrated by an analysis of patients undergoing elective hip replacement between 2010-13 that found over 90% of patients received care within their three nearest hospitals, and for patients that did bypass their nearest hospital they only travelled an additional 5.0km on average to receive care.^12^ Moreover, patient surveys have indicated that most patients prioritise geographical location and convenience as the most important factors when choosing healthcare provider.^13^

- - 1. Independent sector treatment centres

There was consensus among the Labour governments of the 2000s that due to years of underinvestment in healthcare capital and workforce, there was limited capacity in NHS hospitals to clear waiting lists and meet growing demand. One strategy promoted among policy makers was to develop treatment centres that could specialise in high-volume and low complexity elective care procedures, many of which could be done as day cases. They were originally intended to be developed by NHS hospitals, but this model was expanded in 2003 to allow private healthcare providers to develop treatment centres, known as “Independent Sector Treatment Centres (ISTCs)”.^14^ Many ISTCs specialise in one type of procedure, for example cataract surgery. In contrast, private hospitals would provide a broad range of surgical operations and outpatient appointments. By 2010/11, there were 161 ISTCs operating in England.^15^ The policy was perceived as a practical way to significantly increase capacity for elective care within the healthcare system in a relatively short period of time. However, it was also a controversial policy as several stakeholders raised concerns such as the quality of care within ISTCs, the availability of appropriately trained staff, and the potential that ISTCs would engage in risk selection which would leave NHS hospitals with on-average sicker patients and higher costs.^16^

- - 1. Activity-based payments

At the same time, the NHS in England introduced reforms to the hospital reimbursement system.^17^ From 2003, the NHS launched an activity-based payment system for hospitals known as the Payment by Results (PbR). The PbR programme is a national tariff system involving fixed costs for thousands of Healthcare Resource Groups (HRGs) based on the public sector average costs for relevant individual procedures or hospital episodes. Each HRG is refined further when calculating the final reimbursement for each hospital episode based on a range of factors such as patient characteristics and local healthcare labour markets. This system was originally introduced solely for elective care, but was soon expanded to acute, emergency and outpatient care. The PbR system successfully created incentives to improve efficiency,^18^ and also provided a standardised framework that could be used when contracting with private hospitals.^19^ However, there have been criticisms of the PbR reimbursement system including evidence of up-coding by hospitals through a process known as “HRG creep”, ^20^ and an absence of mechanisms to incentivise healthcare quality and patient experiences of care.^17^

- - 1. Greater emphasis on targets

During the early 2000s, there were substantial increases in healthcare spending.^6^ However, there was also increased pressure to ensure value for money for this additional spend of tax-payer monies. In response, the NHS in England became increasingly focused on performance management and implemented a range of targets to reduce waiting times for elective care. The 2000 NHS plan introduced two new targets: 3 months for a first outpatient appointment across all medical and surgical specialities, and 6 months for inpatient treatment.^8^ In 2004, the NHS introduced the 18-week referral-to-treatment target. This changed the way in which waiting times were measured, with waiting times beginning from referral by a GP and only ending once a patient started treatment or was discharged.^21^ Waiting times were routinely published and increasingly became the focus of media attention. Meeting targets was incentivised through penalties – for example, the dismissal of managers of hospitals or local commissioning bodies.^22^ This period of centrally imposed targets and penalties created a culture of mistrust and has been described by policy commentators and academics as the era of “targets and terror”. ^22,23^ The urgency in achieving these targets at both the national and local level provides further insights into why NHS commissioners increasingly looked to private healthcare providers for additional capacity to clear waiting lists.

- - 1. Strengthened regulation

Another important factor which facilitated the introduction of private healthcare providers of publicly funded care was strengthened regulation to ensure that all healthcare providers, irrespective of whether they were NHS or private, met minimum quality of care and patient safety standards. In 2004, the Commission for Healthcare Audit and Inspection (CHAI) was established (replacing the Commission for Health Improvement (CHI)). In acknowledgement that private hospitals were providing an increasing amount of publicly and privately funded care, the mandate of CHAI included regulation of private healthcare providers , by means of registration, inspection, monitoring of complaints, and enforcement activities.^24^ In 2009, CHAI was replaced by the CQC (i.e. the Care Quality Commission), which was an amalgamation of the existing inspectorates responsible for physical healthcare, mental health and social care. The CQC conducts regular inspections and provides ratings for all health and social care providers, both public and private, in England.

- - 1. Structural changes to promote an internal market for healthcare

Alongside the reforms described above to facilitate patient choice, there have been several structural reforms over the last four decades that established and strengthened the internal market for healthcare in England and facilitated the provision of publicly funded care in private hospitals. In the early 1990s, the incumbent Conservative government introduced the 1990 NHS and Community Care Act that created a split between the purchasers (District Health Authorities (DHAs)) and providers of services (NHS Trusts).^25^ The successive Labour government that came to power in 1997 with mandate to abolish the internal market, but retained the separation between purchasers and providers, and the term “purchasing” was replaced by “commissioning” to reframe the relationship between purchasers and providers as working together to improve the quality of healthcare services. Primary Care Trusts (PCTs) were established in 2002, with budgets allocated according to local needs, and responsibility for commissioning NHS services for geographically defined populations.^26^ The reorganisation of DHAs to PCTs was widely understood as costly and disruptive,^27^ and there were no further top-down reorganisations of the NHS until the 2012 Health and Social Care Act was introduced by the Coalition (Conservative and Liberal Democrat) government that came to power in 2010.^28^ The Act replaced PCTs with Clinical Commissioning Groups (CCGs), and included several measures which consolidated and accelerated the internal market for healthcare in England. The most controversial aspect of the Act was “Section 75”, that stated CCGs must protect the right of patients to choose healthcare providers, not engage in anti-competitive behaviour, and introduce competitive tendering for provision of services.^28^ In combination with reforms related to facilitating patient choice, the Health and Social Care Act 2012 created an environment where private healthcare providers could compete for CCG contracts on an equal statutory footing as existing NHS providers.

- - 1. References

1. NICE. What we do. 2024. https://www.nice.org.uk/about/what-we-do (accessed March 26, 2024).
2. CQC. About us. 2024. https://www.cqc.org.uk/about-us (accessed March 26, 2024).
3. NHS Resolution. About. 2024 https://resolution.nhs.uk/about/. (accessed March 26, 2024).
4. BMA. Outsourced: the role of the independent sector in the NHS. 2022. https://www.bma.org.uk/media/5378/bma-nhs-outsourcing-report-march-2022.pdf (accessed March 26, 2024).
5. Murray R. Lessons from the 2000s: the ambition to reduce waits must be matched with patience and realism This content relates to the following topics: 2021. https://www.kingsfund.org.uk/publications/nhs-waiting-times#waiting-times-in-the-1990s-and-2000s (accessed March 26, 2024).
6. Wanless D. Securing our Future Health: Taking a Long-Term View. HM Treasury, 2002.
7. Appleby J, Harrison A, Delvin N. What is the Real Cost of More Patient Choice? 2003. https://www.kingsfund.org.uk/sites/default/files/field/field_publication_file/what-is-real-cost-more-patient-choice-john-appleby-tony-harrison-nancy-devlin-kings-fund-1-june-2003.pdf (accessed March 26, 2024).
8. NHS England. The NHS Plan. 2000. https://www.bsuh.nhs.uk/library/wp-content/uploads/sites/8/2020/09/The-NHS-plan-2000.pdf (accessed March 26, 2024).
9. NHS England. Delivering the NHS Plan: Next Steps on Investment, Next Steps on Reform. 2002. https://webarchive.nationalarchives.gov.uk/ukgwa/20130107105354/http://www.dh.gov.uk/prod_consum_dh/groups/dh_digitalassets/@dh/@en/@ps/documents/digitalasset/dh_118524.pdf (accessed March 26, 2024).
10. Department of Health & Social Care. The NHS Constitution for England. 2015. https://www.gov.uk/government/publications/the-nhs-constitution-for-england/the-nhs-constitution-for-england (accessed March 26, 2024).
11. NHS Confederation. Any Qualified Provider: Discussion paper. 2011. https://www.nhsconfed.org/sites/default/files/2021-05/Any-qualified-provider.pdf (accessed March 26, 2024).
12. Gutacker N, Siciliani L, Moscelli G, Gravelle H. Choice of hospital: Which type of quality matters? J Health Econ 2016; 50: 230–46.
13. Dixon S. Report on the National Patient Choice Survey - February 2010 England. 2010. https://assets.publishing.service.gov.uk/government/uploads/system/uploads/attachment_data/file/216073/dh_117096.pdf (accessed March 26, 2024).
14. Naylor C, Gregory S. Independent sector treatment centres. The King’s Fund. 2009. https://www.kingsfund.org.uk/insight-and-analysis/evidence-and-consultations/independent-sector-treatment-centres (accessed March 26, 2024).
15. Kelly E, Tetlow G. Understanding competition and choice in the NHS: Choosing the place of care. 2012. https://www.nuffieldtrust.org.uk/files/2017-01/choosing-place-of-care-web-final.pdf (accessed March 26, 2024).
16. Pollock AM, Godden S. Independent sector treatment centres: evidence so far. BMJ 2008; 336: 421–4.
17. Grašič K, Mason AR, Street A. Paying for the quantity and quality of hospital care: the foundations and evolution of payment policy in England. Health Econ Rev 2015; 5: 15.
18. Farrar S, Yi D, Sutton M, Chalkley M, Sussex J, Scott A. Has payment by results affected the way that English hospitals provide care? Difference-in-differences analysis. BMJ 2009; 339: b3047.
19. Savva N, Tezcan T, Yıldız Ö. Can yardstick competition reduce waiting times? Manag Sci 2019; 65: 3196–215.
20. Yi D, Pugh E, Farrar S. The Effect of Payment By Results on the HRG Creep in English Hospitals: An Empirical Investigation. 2007. https://papers.ssrn.com/sol3/papers.cfm?abstract_id=993390 (accessed March 26, 2024).
21. Charlesworth A, Watt T, Gardner T. Returning NHS waiting times to 18 weeks for routine treatment: The scale of the challenge pre-COVID-19. 2020. https://www.health.org.uk/publications/long-reads/returning-nhs-waiting-times-to-18-weeks (accessed March 26, 2024).
22. Propper C, Sutton M, Whitnall C, Windmeijer F. Did ‘Targets and Terror’ Reduce Waiting times in England for Hospital Care? The Centre for Market and Public Organisation, University of Bristol, UK, 2007 https://ideas.repec.org/p/bri/cmpowp/07-179.html (accessed March 26, 2024).
23. Bevan G, Hood C. Have targets improved performance in the English NHS? BMJ 2006; 332: 419–22.
24. Commission for Healthcare Audit and Inspection. Our statutory role. 2009. https://webarchive.nationalarchives.gov.uk/ukgwa/20090321145752mp_/http://www.healthcarecommission.org.uk/aboutus/whatwedo/ouraims/ourstatutoryrole.cfm (accessed March 26, 2024).
25. UK Government. National Health Service and Community Care Act 1990. 1990. https://www.legislation.gov.uk/ukpga/1990/19/contents (accessed March 26, 2024).
26. Walshe K, Smith J, Dixon J, et al. Primary care trusts. The BMJ 2004; 329: 871–2.
27. Smith J, Walshe K, Hunter DJ. The “redisorganisation” of the NHS. BMJ 2001; 323: 1262–3.
28. UK Government. Health and Social Care Act. London: Department of Health, 2012. https://www.legislation.gov.uk/ukpga/2012/7/contents (accessed March 26, 2024).

# Supplementary Tables

Table 1: Hip primary OPCS procedure codes and descriptions

| Item | Operation OPCS codes | Description |
| --- | --- | --- |
| H1.1 | W371 | Primary total prosthetic replacement of hip joint using cement |
| H1.2 | W381 | Primary Total Prosthetic Replacement not using cement |
| H1.3 | W391 | Primary total prosthetic replacement of hip joint NEC |
| H1.4 | W431, Z843 | Primary total prosthetic replacement of joint using cement NEC \| Hip Joint |
| H1.5 | W441, Z843 | Primary total prosthetic replacement of joint not using cement NEC \| Hip Joint |
| H1.6 | W451, Z843 | Primary total prosthetic replacement of joint NEC \| Hip Joint |
| H1.7 | W461 | Primary prosthetic replacement of head of femur using cement |
| H1.8 | W471 | Primary prosthetic replacement of head of femur not using cement |
| H1.9 | W481 | Primary prosthetic replacement of head of femur not elsewhere classified |
| H1.10 | W521, Z843 | Primary prosthetic replacement of articulation of bone using cement NEC \| Hip Joint |
| H1.11 | W521, Z756, Z761 | Primary prosthetic replacement of articulation of bone using cement NEC \| Acetabulum \| Head of Femur |
| H1.12 | W531, Z843 | Primary prosthetic replacement of articulation of bone not using cement NEC \| Hip Joint |
| H1.13 | W531, Z756, Z761 | Primary prosthetic replacement of articulation of bone not using cement NEC \| Acetabulum \| Head of Femur |
| H1.14 | W541, Z843 | Primary prosthetic replacement of articulation of bone NEC \| Hip Joint |
| H1.15 | W541, Z756, Z761 | Primary prosthetic replacement of articulation of bone NEC \| Acetabulum \| Head of Femur |
| H1.16 | W581, Z843 | Primary resurfacing arthroplasty of joint \| Hip joint |
| H1.17 | W581, Z902 | Primary resurfacing arthroplasty of joint \| Hip NEC |
| H1.18 | W581, Z756, Z761 | Primary resurfacing arthroplasty of joint \| Acetabulum \| Head of femur |
| H1.19 | W581, W378 | Primary resurfacing arthroplasty of joint \| Other specified total prosthetic replacement of hip joint using cement |
| H1.20 | W581, W388 | Primary resurfacing arthroplasty of joint \| Other specified total prosthetic replacement of hip joint not using cement |
| H1.21 | W581, W391 | Primary resurfacing arthroplasty of joint \| Primary total prosthetic replacement of hip joint NEC |
| H1.22 | W581, W398 | Primary resurfacing arthroplasty of joint \| Other specified other total prosthetic replacement of hip joint |
| H1.23 | W581, W461, Z756 | Primary resurfacing arthroplasty of joint \| Primary prosthetic replacement of head of femur using cement \| Acetabulum |
| H1.24 | W581, W471, Z756 | Primary resurfacing arthroplasty of joint \| Primary prosthetic replacement of head of femur not using cement \| Acetabulum |
| H1.25 | W581, W481, Z756 | Primary resurfacing arthroplasty of joint \| Primary prosthetic replacement of head of femur NEC \| Acetabulum |
| H1.26 | W931 | Primary hybrid prosthetic replacement of hip joint using cemented acetabular component |
| H1.27 | W941 | Primary hybrid prosthetic replacement of hip joint using cemented femoral component |
| H1.28 | W951 | Primary hybrid prosthetic replacement of hip joint using cement NEC |

Source: NJR 2023 https://www.njrcentre.org.uk/healthcare-providers/manuals-and-training/

Table 2: Knee primary OPCS procedure codes and descriptions

| Item | Operation OPCS codes | Description |
| --- | --- | --- |
| K1.1 | O181 | Primary hybrid prosthetic replacement of knee joint using cement |
| K1.2 | W401 | Primary total prosthetic replacement of knee joint using cement |
| K1.3 | W411 | Primary total prosthetic replacement of knee joint not using cement |
| K1.4 | W421 | Primary total prosthetic replacement of knee joint NEC |
| K1.5 | W431, Z844 | Primary total prosthetic replacement of joint using cement NEC \|  Patellofemoral joint |
| K1.6 | W431, Z845 | Primary total prosthetic replacement of joint using cement NEC \|  Tibiofemoral joint |
| K1.7 | W431, Z846 | Primary total prosthetic replacement of joint using cement NEC \| Knee  joint |
| K1.8 | W441, Z844 | Primary total prosthetic replacement of joint not using cement NEC \|  Patellofemoral joint |
| K1.9 | W441, Z845 | Primary total prosthetic replacement of joint not using cement NEC \|  Tibiofemoral joint |
| K1.10 | W441, Z846 | Primary total prosthetic replacement of joint not using cement NEC \| Knee joint |
| K1.11 | W451, Z844 | Primary total prosthetic replacement of joint NEC \| Patellofemoral joint |
| K1.12 | W451, Z845 | Primary total prosthetic replacement of joint NEC \| Tibiofemoral joint |
| K1.13 | W451, Z846 | Primary total prosthetic replacement of joint NEC \| Knee joint |
| K1.14 | W521, Z844 | Primary prosthetic replacement of articulation of bone using cement  NEC \| Patellofemoral joint |
| K1.15 | W521, Z845 | Primary prosthetic replacement of articulation of bone using cement  NEC \| Tibiofemoral joint |
| K1.16 | W521, Z846 | Primary prosthetic replacement of articulation of bone using cement  NEC \| Knee joint |
| K1.17 | W531, Z844 | Primary prosthetic replacement of articulation of bone not using  cement NEC \| Patellofemoral joint |
| K1.18 | W531, Z845 | Primary prosthetic replacement of articulation of bone not using  cement NEC \| Tibiofemoral joint |
| K1.19 | W531, Z846 | Primary prosthetic replacement of articulation of bone not using  cement NEC \| Knee joint |
| K1.20 | W541, Z844 | Primary prosthetic replacement of articulation of bone NEC \| Patellofemoral joint |
| K1.21 | W541, Z845 | Primary prosthetic replacement of articulation of bone NEC \|  Tibiofemoral joint |
| K1.22 | W541, Z846 | Primary prosthetic replacement of articulation of bone NEC \| Knee joint |
| K1.23 | W581, Z844 | Primary resurfacing arthroplasty of joint \| Patellofemoral joint |
| K1.24 | W581, Z845 | Primary resurfacing arthroplasty of joint \| Tibiofemoral joint |
| K1.25 | W581, Z846 | Primary resurfacing arthroplasty of joint \| Knee joint |

Source: NJR 2023 https://www.njrcentre.org.uk/healthcare-providers/manuals-and-training/

Table 3: ICD-10 codes used for identification of adverse events

| Adverse Event | ICD-10 codes | Exclusions |
| --- | --- | --- |
| Adverse drug reaction | Y400-Y599  Y601-Y603  Y621-Y623  Y630-Y639  Y640-Y649  Y650  Y651  T881  T886  T887 |  |
| Infection | T814  T793  T826-T827  T835  T836  T845-T847  T857  T802  T880  Y95  N390  J120-J189  J200-J22  A400-A419  R578  T811 | Primary diagnosis=T793, T826-T827,T835-T836, T845-T847, T857, T802,T880, Y9, A40-A41,R578, T81  Length of stay <4 |
| Pressure ulcer | L89 | Primary diagnosis=L8  G81, G82, Q05, G931  Length of stay <5 |
| Venous Thromboembolism | I260  I269  I801  I802  I803  I808  I809  I828  I829 | Primary diagnosis= I260, I269, I801, I802, I803, I808, I809, I828, I829  Primary operation code=L791, L792, L798 |

Source: Friebel et al 2021, Romano et al 2009

Table 4: Missing data

|  | NHS hospital | | Private Hospital | |
| --- | --- | --- | --- | --- |
| Variable | Present | % of records | Present | % of records |
| In-hospital mortality | 362,659 | 100.000% | 169,232 | 100.000% |
| Emergency readmission | 362,659 | 100.000% | 169,232 | 100.000% |
| Hospital transfer | 362,659 | 100.000% | 169,232 | 100.000% |
| Length of stay | 362,659 | 100.000% | 169,232 | 100.000% |
| Hospital associated infection | 362,659 | 100.000% | 169,232 | 100.000% |
| Adverse drug reaction | 362,659 | 100.000% | 169,232 | 100.000% |
| Pressure ulcer | 362,659 | 100.000% | 169,232 | 100.000% |
| Venous thromboembolism | 362,659 | 100.000% | 169,232 | 100.000% |
| Gender | 362,652 | 99.998% | 169,204 | 99.983% |
| Age | 362,659 | 100.000% | 169,232 | 100.000% |
| Index of Multiple Deprivation | 356,553 | 98.316% | 168,845 | 99.771% |
| Distance to nearest private hospital | 356,553 | 98.316% | 168,845 | 99.771% |
| Distance to nearest NHS hospital | 356,553 | 98.316% | 168,845 | 99.771% |
| Lower layer super output area 2011 | 356,553 | 98.316% | 168,845 | 99.771% |
| Charlson Comobordiy Index | 362,659 | 100.000% | 169,232 | 100.000% |
| Discharge Date | 362,659 | 100.000% | 169,232 | 100.000% |
| Pre-operative patient reported outcome measure (until March 2018) | 159,078 | 63.509% | 72,360 | 62.408% |
| Healthcare Resource Group | 362,659 | 100.000% | 169,232 | 100.000% |
| Primary diagnostic code | 362,659 | 100.000% | 169,232 | 100.000% |
| Primary procedural code | 362,659 | 100.000% | 169,232 | 100.000% |
| Discharge destination | 362,659 | 100.000% | 169,232 | 100.000% |
| Total records | 362,659 | 100.000% | 169,232 | 100.000% |

Table 5: Hospital Type and Volume between 2016-2019

|  | Number of sites | Volumes |
| --- | --- | --- |
| **NHS hospitals** | 310 | 362,659 |
|  |  |  |
| *-NHS Hospitals (exc treatment centres (244)* | 305 | 347,192 |
| *-NHS Treatment Centre (5)* | 5 | 15,467 |
| **Private hospital** | 185 | 169,232 |
|  |  |  |
| *-Private Hospital (exc ISTC)* | 158 | 130,295 |
| *-ISTC* | 27 | 38,937 |
|  |  |  |
| *-Private hospital*  *for-profit* | 150 | 135,223 |
| *-Private hospital*  *not-for-profit* | 35 | 33,435 |
| **All hospitals (415)** | 495 | 531,891 |

Table 6: HRGs for NHS and Private hospitals

| HRG4 Code | Name | NHS Hospital | % | Private Hospital | % | Total | % |
| --- | --- | --- | --- | --- | --- | --- | --- |
| HB11 | Major Hip Procedures for non-trauma, Category 2 | 17,562 | 4.84 | 7,290 | 4.31% | 24,852 | 4.67 |
| HB12 | Major Hip Procedures for non-trauma, Category 1 | 45,142 | 12.45 | 20,633 | 12.19 | 65,775 | 12.37 |
| HB21 | Major Knee Procedures for Non-Trauma, Category 2 | 58,104 | 16.02 | 20,888 | 12.34 | 78,992 | 14.85 |
| HB23 | Intermediate Knee Procedures for non-trauma | 175 | 0.05 | 20 | 0.01 | 195 | 0.04 |
| HN12 | Very Major Hip Procedures for non-trauma | 98,545 | 27.17 | 47,267 | 27.93 | 145,812 | 27.41 |
| HN22 | Very Major Knee Procedures for Non-Trauma | 115,701 | 31.9 | 56,895 | 33.62 | 172,596 | 32.45 |
| HN23 | Major Knee Procedures for Non-Trauma | 43 | 0.01 | 11 | 0.01 | 54 | 0.01 |
| HN80 | Very Complex, Hip or Knee Procedures for non-trauma | 324 | 0.09 | 45 | 0.03 | 369 | 0.07 |
| HN81 | Complex, Hip or Knee Procedures for non-trauma | 6,667 | 1.84 | 827 | 0.49 | 7,494 | 1.41 |
| HR04 | Reconstruction Procedures Category 3 | 568 | 0.16 | 43 | 0.03 | 611 | 0.11 |
| HR05 | Reconstruction Procedures Category 2 | 19,725 | 5.44 | 15,104 | 8.93 | 34,829 | 6.55 |
| UZ01 | Data Invalid for Grouping | 103 | 0.03 | 209 | 0.12 | 312 | 0.06 |
| Total |  | 362,659 |  | 169,232 |  | 531,891 |  |

Table 7: Results of first stage regression for 2SLS IV analysis

|  | IV First stage  (D_NHS_ – D_private_ IV ) | P  value | IV First stage  (D_NHS,_ D_private_ IV ) | P  value |
| --- | --- | --- | --- | --- |
| D_NHS_ – D_private_ | 0.007 (0.007, 0.007) | <0.001 |  |  |
| D_NHS_ |  |  | 0.010 (0.009, 0.010) | <0.001 |
| D_private_ |  |  | -0.006 (-0.007, -0.006) | <0.001 |
| Age 18–40 | — |  | - |  |
| Age 41–60 | 0.060 (0.029, 0.091) | <0.001 | 0.058 (0.027, 0.089) | <0.001 |
| Age 61–80 | 0.222 (0.195, 0.248) | <0.001 | 0.216 (0.190, 0.243) | <0.001 |
| Age >80 | 0.161 (0.134, 0.188) | <0.001 | 0.156 (0.129, 0.182) | <0.001 |
| Gender | -0.012 (-0.014, -0.009) | <0.001 | 0.011 (-0.013, 0.009) | <0.001 |
| IMD 1 | — |  | - |  |
| IMD 2 | 0.005 (0.001, 0.009) | 0.025 | 0.006 (0.001, 0.010) | 0.010 |
| IMD 3 | 0.013 (0.008, 0.017) | <0.001 | 0.012 (0.007, 0.016) | <0.001 |
| IMD 4 | 0.059 (0.055, 0.063) | <0.001 | 0.055 (0.051, 0.059) | <0.001 |
| IMD 5 | 0.064 (0.060, 0.068) | <0.001 | 0.062 (0.058, 0.066) | <0.001 |
| CCI 0 | — |  | - |  |
| CCI 1 | -0.075 (-0.078, -0.072) | <0.001 | -0.075 (-0.076, -0.072) | <0.001 |
| CCI 2 | -0.105 (-0.109, -0.101) | <0.001 | -0.105 (-0.110, -0.101) | <0.001 |
| CCI 3 | -0.136 (-0.143, -0.129) | <0.001 | -0.136 (-0.143, -0.129) | <0.001 |
| CCI 4 | -0.179 (-0.191, -0.166) | <0.001 | -0.178 (-0.191, -0.166) | <0.001 |
| CCI 5 | -0.210 (-0.234, -0.186) | <0.001 | -0.209 (-0.233, -0.185) | <0.001 |
| CCI 6 | -0.202 (-0.228, -0.177) | <0.001 | -0.201 (-0.227, -0.176) | <0.001 |
| Constant | 0.198 (0.170, 0.227) |  | 0.174 (0.145, 0.202) |  |
| Observations | 526,266 |  | 526,266 |  |
| R^2^ | 0.0532 |  | 0.0554 |  |
| F Stat | 9404.40 |  | 5338.27 |  |

IMD: index of multiple deprivation, CCI: Charlson Comorbidity Index, Q: quintile (quintile 1 = most deprived, quintile 5 = least deprived).

*Table 8: Results of alternative specification of IV model (distance to nearest NHS hospital, and distance nearest private hospital as two separate instruments)*

|  | Instrumental variable analyses  (1) |
| --- | --- |
| In-hospital mortality  P value  R^2^:  Endog test p value:  Overid test p value: | -0.0009 (-0.014, -0.0007)  0.0316  0.4237  0.9639  0.0034 |
| Readmission  P value  R^2^:  Endog test p value:  Overid test p value: | -0.0197 (-0.0320, -0.0073)  0.0018  0.2335  0.8782  <0.0001 |
| Hospital transfer  P value  R^2^:  Endog test p value:  Overid test p value: | 0.0084 (-0.0079, 0.0248)  0.3126  0.1704  <0.0001  <0.0001 |
| Pre-op LOS  P value  R^2^:  Endog test p value:  Overid test p value: | 0.0631 (0.0006, 0.1255)  0.0477  0.5221  <0.0001  <0.0001 |
| Post-op LOS  P value  R^2^:  Endog test p value:  Overid test p value: | 0.2001 (0.1032, 0.2969)  0.0001  0.3461  <0.0001  <0.0001 |
| HAI  P value  R^2^  Endog test p value:  Overid test p value: | -0.0089 (-0.0121, -0.0056)  0.0000  0.6670  0.3534  <0.0001 |
| Adverse drug reaction  P value  R^2^  Endog test p value:  Overid test p value: | -0.0008 (-0.0039, 0.0022)  0.5903  0.0963  0.0107  0.0002 |
| Pressure ulcer  P value  R^2^  Endog test p value:  Overid test p value: | -0.0015 (-0.0026, -0.004)  0.0062  0.2245  0.8436  0.0006 |
| Venous thrombo-embolism  P value  R^2^  Endog test p value:  Overid test p value: | -0.0013 (-0.0029, 0.0004)  0.1328  0.2414  0.2386  0.0068 |
| 1st-stage F stat:  Observations: | 5339.37  525,361 |

Endog test: Hausman endogeneity test, Overid test: Sargan-Hansen overidentification test, HAI: healthcare-associated infection, LOS: length of stay

Table 9: Results of OLS and IV models for all NHS and private hospitals when including PROMs as covariate

|  | Probit regression with case-mix adjustment | Instrumental variable analyses |
| --- | --- | --- |
| In-hospital mortality  P value  R^2^:  Endog test p value: | -0.0008 (-0.0012,-0.0004)  <0.0001  0.0157 | 0.0002 (-0.0018, 0.0022)  0.8264  0.0005  0.3005 |
| Readmission  P value  R^2^:  Endog test p value: | -0.0142 (-0.0156,-0.0128)  <0.0001  0.1186 | 0.0117 (0.0043, 0.0190)  0.0020  0.0014  0.0016 |
| Hospital Transfer  P value  R^2^:  Endog test p value: | -0.0048 (-0.0059,-0.0037)  <0.0001  0.0918 | 0.0237 (-0.0283, 0.0757)  0.3711  0.4154  <0.0001 |
| Pre-op LOS > 0 days  P-value  R^2^:  Endog test p value: | 0.0608 (0.0397,0.0818)  <0.0001  0.5612 | 0.2943 (0.1894, 0.3993)  <0.0001  0.8246  <0.0001 |
| Post-op LOS > 2 days  P-value  R^2^:  Endog test p value: | -0.1352 (-0.1727,-0.0978)  <0.0001  0.3884 | 0.2943 (0.1894, 0.3993)  <0.0001  0.5339  <0.0001 |
| HAI  P value  R^2^  Endog test p value: | -0.0100 (-0.0108, -0.0091)  <0.0001  0.1523 | -0.0038 (-0.0081, 0.0006)  0.0918  0.2997  0.0259 |
| Adverse drug reaction  P value  R^2^  Endog test p value: | -0.0052 (-0.0061, -0.0043)  <0.0001  0.2717 | 0.0054 (-0.0011, 0.0119)  0.1007  0.2049  0.0012 |
| Pressure Ulcer  P value  R^2^  Endog test p value: | -0.0018 (-0.0022, -0.0013)  <0.0001  0.0568 | 0.0014 (-0.0011 0.0039)  0.2725  0.0192  0.0992 |
| Venous thrombo-embolism  P value  R^2^  Endog test p value: | -0.0028 (-0.0033, -0.0023)  <0.0001  0.2856 | 0.0003 (-0.0033, 0.0040)  0.8596  0.0026  0.0967 |
| 1st stage F stat:  Observations: | 229,507 | 3880.52  229,507 |

Endog test: Hausman endogeneity test, HAI: healthcare-associated infection, LOS: length of stay. The endogeneity tests for the London analysis were run without clustering at the HRG level, as there were too few clusters to run this test.

Table 10: Results of OLS and IV models for all NHS and private hospitals for London subanalysis

|  | Probit regression with case-mix adjustment | Instrumental variable analyses |
| --- | --- | --- |
| In-hospital mortality  P value  R^2^:  Endog test p value: | -0.0005 (-0.0016,0.0006)  0.4318  0.0032 | - |
| Readmission  P value  R^2^:  Endog test p value: | -0.0168 (-0.0246,-0.0091)  <0.0001  0.0662 | 0.0444 (-0.1228,0.0340)  0.2674  0.3519  0.4585 |
| Hospital Transfer  P value  R^2^:  Endog test p value: | -0.0139 (-0.0165,-0.0114)  <0.0001  0.1328 | -0.0190 (-0.0585,0.0204)  0.3447  0.6675  0.7036 |
| Pre-op LOS > 0 days  P-value  R^2^:  Endog test p value: | 0.0489 (-0.0078,0.1057)  0.0916  0.2044 | 0.1121 (-0.0011,0.2253)  0.0523  0.5218  0.0192 |
| Post-op LOS > 2 days  P-value  R^2^:  Endog test p value: | -0.1538 (-0.2584,-0.0492)  0.0039  0.3912 | 0.1689 (-0.0208,0.3587)  0.0810  0.2007  <0.0001 |
| HAI  P value  R^2^  Endog test p value: | -0.0122 (-0.0152, -0.0091)  <0.0001  0.0348 | -0.0404 (-0.0630,-0.0177)  0.0005  0.8489  0.1658 |
| Adverse drug reaction  P value  R^2^  Endog test p value: | -0.0142 (-0.0192, -0.0092)  <0.0001  0.1999 | 0.0440 (-0.0223, 0.1103)  0.1935  0.7022  0.0016 |
| Pressure Ulcer  P value  R^2^  Endog test p value: | -0.0010 (-0.0022, 0.0002)  0.1111  0.0157 | - |
| Venous thrombo-embolism  P value  R^2^  Endog test p value: | -0.0061 (-0.0087, -0.0034)  <0.0001  0.2321 | -0.0047 (-0.0276, 0.0182)  0.6901  0.3523  0.9931 |
| 1st stage F stat:  Observations: | 41,801 | 496.664  41,799 |

Endog test: Hausman endogeneity test, HAI: healthcare-associated infection, LOS: length of stay. The endogeneity tests for the London analysis were run without clustering at the HRG level, as there were too few clusters to run this test.

Table 11: Results of OLS and IV models for NHS treatment centres versus Independent Sector Treatment Centres (ISTCs)

|  | Probit regression with case-mix adjustment | Instrumental variable analyses |
| --- | --- | --- |
| In-hospital mortality  P-value  R^2^:  Endog test p value: | - | - |
| Readmission  P-value  R^2^:  Endog test p value: | -0.0068 (-0.0149,0.0012)  0.0955  0.0551 | 0.1849 (0.0565,0.3132)  0.0047  0.7918  0.0007 |
| Hospital Transfer  P-value  R^2^:  Endog test p value: | -0.0027 (-0.0045,-0.0008)  0.0057  0.2290 | 0.0092 (-0.0454,0.0638)  0.7415  0.4450  0.4520 |
| Pre-op LOS > 0 days  P-value  R^2^:  Endog test p value: | -0.0060 (-0.0075,-0.0046)  <0.0001  0.4858 | 0.0002 (-0.0178,0.0181)  0.9868  0.0027  0.8098 |
| Post-op LOS > 2 days  P-value  R^2^:  Endog test p value: | -0.0984 (-0.1401,-0.0567)  <0.0001  0.2003 | -0.3967 (-0.6329,-0.1605)  0.0010  0.8275  <0.0001 |
| HAI  P-value  R^2^  Endog test p value: | -0.0018 (-0.0023, -0.0014)  <0.0001  0.0359 | -0.3065 (-0.4706, -0.1424)  0.0003  0.9789  0.0228 |
| Adverse drug Reaction  P-value  R^2^  Endog test p value: | -0.0119 (-0.0143, -0.0094)  <0.0001  0.7970 | -0.2882 (-0.4000, -0.1765)  <0.0001  0.9827  0.0173 |
| Pressure Ulcer  P-value  R^2^  Endog test p value: | -0.0009 (-0.0018, 0.0001)  0.0530  0.0578 | 0.0173 (-0.0622, 0.0968)  0.5259  0.7389  0.3862 |
| Venous thrombo-Embolism  P-value  R^2^  Endog test p value: | -0.0018 (-0.0024, -0.0013)  <0.0001  0.5618 | -0.2482 (-0.8518, 0.3552)  0.4201  0.9708  0.0788 |
| 1st stage F stat:  Observations: | 54,143 | 166.696  54,143 |

Endog test: Hausman endogeneity test, HAI: healthcare-associated infection, LOS: length of stay. The endogeneity tests were run without clustering at the HRG level, as there were too few clusters to run this test.

Table 12: Results of OLS and IV models for NHS Acute Hospital versus Private Hospitals (excluding ISTCs)

|  | Probit regression with case-mix adjustment | Instrumental variable analyses |
| --- | --- | --- |
| In-hospital mortality  P-value  R^2^:  Endog test p value: | -0.0008 (-0.0010, -0.007)  <0.0001  0.0344 | -0.0002 (-0.0010, 0.0006)  0.6693  0.0523  0.2265 |
| Readmission  P-value  R^2^:  Endog test p value: | -0.0192 (-0.0205, -0.0179)  <0.0001  0.1439 | 0.0024 (-0.0122,0.0171)  0.7465  0.0081  0.0004 |
| Hospital Transfer  P-value  R^2^:  Endog test p value: | -0.0076 (-0.0084, -0.0069)  <0.0001  0.1434 | 0.0098 (-0.0094,0.0290)  0.3177  0.1858  <0.0001 |
| Pre-op LOS > 0 days  P-value  R^2^:  Endog test p value: | 0.0436 (0.0130, 0.0742)  0.0052  0.3318 | 0.0673 (-0.0076,0.1422)  0.0782  0.5130  <0.0001 |
| Post-op LOS > 2 days  P-value  R^2^:  Endog test p value: | -0.1432 (-0.1922,-0.0943)  <0.0001  0.3693 | 0.2721 (0.1716,0.3726)  <0.0001  0.4893  <0.0001 |
| HAI  P-value  R^2^  Endog test p value: | -0.0125 (-0.0132, -0.0117)  <0.0001  0.1139 | -0.0053 (-0.0082,-0.0024)  0.0004  0.3765  0.0008 |
| Adverse drug reaction  P-value  R^2^  Endog test p value: | -0.0049 (-0.0057, 0.0042)  <0.0001  0.3111 | -0.0014 (-0.0056,0.0027)  0.5013  0.1711  0.0644 |
| Pressure Ulcer  P-value  R^2^  Endog test p value: | -0.0018 (-0.0022, -0.0014)  <0.0001  0.0760 | -0.0002 (-0.0017,0.0013)  0.7988  0.0261  0.2192 |
| Venous thrombo-embolism  P-value  R^2^  Endog test p value: | -0.0024 (-0.0030, -0.0019)  <0.0001  0.2729 | -0.0003 (-0.0017,0.0023)  0.7893  0.0038  0.0543 |
| 1st stage F stat:  Observations: | 471,146 | 8089.12  471,144 |

Endog test: Hausman endogeneity test, HAI: healthcare-associated infection, LOS: length of stay. The endogeneity tests were run without clustering at the HRG level, as there were too few clusters to run this test.

Table 13: Results of OLS and IV Models for NHS hospitals versus For-Profit Private Hospitals

|  | Probit regression with case-mix adjustment | Instrumental variable analyses |
| --- | --- | --- |
| In-hospital mortality  P-value  R^2^:  Endog test p value: | -0.0009 (-0.0010,-0.0007)  <0.0001  0.0330 | -0.0004 (-0.0012,0.0004)  0.3259  0.1610  0.2758 |
| Readmission  P-value  R^2^:  Endog test p value: | -0.0178 (-0.0187,-0.0169)  <0.0001  0.1276 | -0.0018 (-0.0177,0.0142)  0.8269  0.0244  0.0096 |
| Hospital Transfer  P-value  R^2^:  Endog test p value: | -0.0123 (-0.0143,-0.0102)  <0.0001  0.1918 | 0.0017 (-0.0120,0.0154)  0.8104  0.0003  <0.0001 |
| Pre-op LOS > 0 days  P-value  R^2^:  Endog test p value: | 0.0411 (0.0117,0.0706)  0.0062  0.3249 | 0.0614 (-0.0151,0.1380)  0.1158  0.4940  <0.0001 |
| Post-op LOS > 2 days  P-value  R^2^:  Endog test p value: | -0.1182 (-0.1675,-0.0688)  <0.0001  0.2941 | 0.3238 (0.2367,0.4109)  <0.0001  0.6119  <0.0001 |
| HAI  P-value  R^2^  Endog test p value: | -0.0118 (-0.0126, -0.0109)  <0.0001  0.1087 | -0.0055 (-0.0086, -0.0024)  0.0006  0.3983  0.0028 |
| Adverse drug reaction  P-value  R^2^  Endog test p value: | -0.0047 (-0.0052, -0.0043)  <0.0001  0.3024 | 0.0036 (-0.0028, 0.0100)  0.2748  0.1801  0.0003 |
| Pressure Ulcer  P-value  R^2^  Endog test p value: | -0.0016 (-0.0018, -0.0013)  <0.0001  0.0631 | -0.0001 (-0.0015, 0.0013)  0.8709  0.0145  0.3065 |
| Venous thrombo-embolism  P-value  R^2^  Endog test p value: | -0.0027 (-0.0031, -0.0020)  <0.0001  0.3043 | 0.0000 (-0.0018, 0.0018)  0.9829  0.0142  0.0608 |
| 1st stage F stat:  Observations: | 492,027 | 7163.74  492,025 |

HAI: healthcare-associated infection, LOS: length of stay.

Table 14: Results of OLS and IV Models for NHS hospitals versus Not-For-Profit Private Hospitals

|  | Probit regression with case-mix adjustment | Instrumental variable analyses |
| --- | --- | --- |
| In-hospital mortality  P-value  R^2^:  Endog test p value: | -0.0009 (-0.0017, -0.0000)  0.0393  0.0120 | 0.0011 (-0.0034,0.0056)  0.6326  0.0724  0.3392 |
| Readmission  P-value  R^2^:  Endog test p value: | -0.0221 (-0.0256, -0.0185)  <0.0001  0.0761 | 0.0265 (-0.0217,0.0747)  0.2814  0.0404  0.0035 |
| Hospital Transfer  P-value  R^2^:  Endog test p value: | 0.0011 (-0.0023, -0.0000)  0.0454  0.01035 | 0.08333 (-0.0312, 0.1979)  0.1539  0.7210  <0.0001 |
| Pre-op LOS > 0 days  P-value  R^2^:  Endog test p value: | -0.0279 (-0.0309, -0.0249)  <0.0001  0.1762 | -0.2324 (-0.3174,-0.1475)  <0.0001  0.9632  <0.0001 |
| Post-op LOS > 2 days  P-value  R^2^:  Endog test p value: | -0.1341 (-0.1720,-0.0961)  <0.0001  0.1845 | 0.8499 (0.7214,0.9785)  <0.0001  0.8273  <0.0001 |
| HAI  P-value  R^2^  Endog test p value: | -0.0139 (-0.0151, -0.0126)  <0.0001  0.0454 | 0.0041 (-0.0091,0.0172)  0.5458  0.0098  0.0042 |
| Adverse drug reaction  P-value  R^2^  Endog test p value: | -0.0071 (-0.0087, 0.0056)  <0.0001  0.1786 | -0.0010 (-0.01271,0.0106)  0.8597  0.0407  0.0860 |
| Pressure Ulcer  P-value  R^2^  Endog test p value: | -0.0028 (-0.0033, -0.0022)  <0.0001  0.0364 | 0.0014 (-0.036,0.0064)  0.5955  0.0092  0.2892 |
| Venous thrombo-embolism  P-value  R^2^  Endog test p value: | -0.0035 (-0.0046, -0.0023)  <0.0001  0.1720 | 0.0053 (-0.0035,0.0142)  0.2338  0.2554  0.0605 |
| 1st stage F stat:  Observations: | 389,891 | 2968.95  389,889 |

Endog test: Hausman endogeneity test, HAI: healthcare-associated infection, LOS: length of stay. HAI: healthcare-associated infection, LOS: length of stay.

Table 15: Results of nearest neighbour propensity score matching for primary analysis

|  | Hip Replacement | | Knee replacement | |
| --- | --- | --- | --- | --- |
|  | ATT | P-value | ATT | P-value |
| In-hospital mortality | -0.0003  (-0.0005,-0.0001) | <0.001 | -0.0004  (-0.0006,-0.0002) | <0.001 |
| Emergency readmission | -0.0163  (-0.0195, -0.0131) | <0.001 | -0.0176  (-0.0206, -0.0146) | <0.001 |
| Hospital transfer | -0.0062  (-0.0074, -0.0051) | <0.001 | -0.0051  (-0.0061, -0.0042) | <0.001 |
| Pre-operative LOS >0 days | 0.0354  (0.0330, 0.0378) | <0.001 | 0.0392  (0.0370, 0.0415) | <0.001 |
| Post-operative LOS >2 days | -0.1191  (-0.1265, -0.1117) | <0.001 | -0.1336  (-0.1387, -0.1284) | <0.001 |
| HAI | -0.0052  (-0.0059, -0.0044) | <0.001 | -0.0060  (-0.0068, -0.0053) | <0.001 |
| Adverse drug reaction | -0.0044  (-0.0053,-0.0034) | <0.001 | -0.0041  (-0.0051,-0.0032) | <0.001 |
| Pressure ulcer | -0.0013  (-0.0018, -0.0008) | <0.001 | -0.0011  (-0.0015, -0.0006) | <0.001 |
| Venous thromboembolism | -0.0011  (-0.0017, -0.0006) | <0.001 | -0.0025  (-0.0033, -0.0018) | <0.001 |
| Observations | 239,535 |  | 285,824 |  |

Table 16: Quality of covariate matching for primary analysis

| Hip Replacement | | | | | | |
| --- | --- | --- | --- | --- | --- | --- |
|  |  | Before Matching | | After Matching | |  |
|  | Mean Treated | Mean Control | Mean Difference | Mean Control | Mean Difference | Bias Percentage Reduction (%) |
| Age | 68.476 | 68.943 | -0.4670 | 68.505 | -0.0290 | 93.9 |
| Gender | 0.60411 | 0.59830 | 0.0058 | 0.61670 | -0.0126 | -116.9 |
| IMD Quintile | 3.4019 | 3.1742 | 0.2277 | 3.4109 | -0.0090 | 96.1 |
| CCI | 0.40443 | 0.64287 | -0.2384 | 0.40824 | -0.0038 | 98.4 |
| Knee Replacement | | | | | | |
| Age | 69.085 | 69.589 | -0.5040 | 69.264 | -0.1790 | 64.6 |
| Gender | 0.55227 | 0.57542 | -0.0232 | 0.56517 | -0.0129 | 44.4 |
| IMD Quintile | 3.3129 | 3.313 | -0.0001 | 3.3059 | 0.0070 | 96.9 |
| CCI | 0.45779 | 0.66676 | -0.2090 | 0.45374 | 0.0041 | 98.1 |

IMD: Index of multiple deprivation (quintile 1 = most deprived, quintile 5 = least deprived), CCI: Charlson Comorbidity Index

Table 17: Results of nearest neighbour propensity score matching when including PROMs

|  | Hip Replacement | | Knee replacement | |
| --- | --- | --- | --- | --- |
|  | ATT | P-value | ATT | P-value |
| In-hospital mortality | -0.0003  (-0.0007,-0.0000) | 0.059 | -0.0003  (-0.0006,0.0000) | 0.052 |
| Emergency readmission | -0.0144  (-0.0191, -0.0098) | <0.001 | -0.0157  (-0.0204, -0.0111) | <0.001 |
| Hospital transfer | -0.0044  (-0.0057, -0.0032) | <0.001 | -0.0035  (-0.0049, -0.0021) | <0.001 |
| Pre-operative LOS >0 days | 0.0738  (0.0701, 0.0775) | <0.001 | 0.0712  (0.0678, 0.0746) | <0.001 |
| Post-operative LOS >2 days | -0.1337  (-0.1427, -0.1246) | <0.001 | -0.1431  (-0.1519, -0.1344) | <0.001 |
| HAI | -0.0043  (-0.0054, -0.0031) | <0.001 | -0.0053  (-0.0064, -0.0042) | <0.001 |
| Adverse drug reaction | -0.0042  (-0.0056,-0.0028) | <0.001 | -0.0039  (-0.0051,-0.0026) | <0.001 |
| Pressure ulcer | -0.0014  (-0.0022, -0.0006) | <0.001 | -0.0009  (-0.0015, -0.0003) | 0.003 |
| Venous thromboembolism | -0.0016  (-0.0025, -0.0008) | <0.001 | -0.0023  (-0.0032, -0.0013) | <0.001 |
| Observations | 107,664 |  | 121,815 |  |

Table 18: Quality of covariate matching when including PROMs

| Hip Replacement | | | | | | |
| --- | --- | --- | --- | --- | --- | --- |
|  |  | Before Matching | | After Matching | |  |
|  | Mean Treated | Mean Control | Mean Difference | Mean Control | Mean Difference | Bias Percentage Reduction (%) |
| Age | 68.314 | 68.620 | -0.3060 | 68.314 | 0.0000 | 99.8 |
| Gender | 0.60462 | 0.60044 | 0.0042 | 0.60534 | -0.0007 | 81.5 |
| IMD Quintile | 3.4223 | 3.1926 | 0.2297 | 3.4196 | 0.0027 | 98.8 |
| CCI | 0.37063 | 0.66676 | -0.2961 | 0.3658 | 0.0048 | 97.9 |
| PROMs score | 19.039 | 16.582 | 2.4570 | 18.994 | 0.0450 | 98.2 |
| Knee Replacement | | | | | | |
| Age | 69.247 | 69.693 | -0.4460 | 69.247 | 0.0000 | 100.0 |
| Gender | 0.55257 | 0.57893 | -0.0264 | 0.55271 | -0.0001 | 99.4 |
| IMD Quintile | 3.3288 | 3.0985 | 0.2303 | 3.3297 | -0.0009 | 99.6 |
| CCI | 0.43546 | 0.64698 | -0.2115 | 0.43032 | 0.0051 | 97.6 |
| PROMs score | 20.284 | 17.808 | 2.4760 | 20.313 | -0.0290 | 98.8 |

IMD: Index of multiple deprivation (quintile 1 = most deprived, quintile 5 = least deprived), CCI: Charlson Comorbidity Index. PROMs score is pre-operative Oxford Hip and Knee score.
